# Sclerostin in Excessive Drinkers: Relationships with Liver Function and Body Composition

**DOI:** 10.3390/nu14132574

**Published:** 2022-06-22

**Authors:** Candelaria Martín González, Camino María Fernández Rodríguez, Pedro Abreu González, Alen García Rodríguez, Julio César Alvisa Negrín, Elisa Cabañas Perales, Lourdes González Navarrete, Víctor Eugenio Vera Delgado, Paula Ortega Toledo, Emilio González Reimers

**Affiliations:** 1 Departamento de Medicina Interna, Universidad de La Laguna, Servicio de Medicina Interna, Hospital Universitario de Canarias, Tenerife, Canary Islands, 38320 La Laguna, Spain; caminoferro@gmail.com (C.M.F.R.); alengarciarodriguez@gmail.com (A.G.R.); jcalvisa@yahoo.es (J.C.A.N.); ecperales1994@gmail.com (E.C.P.); lourdes.gleznav@gmail.com (L.G.N.); victoringui@gmail.com (V.E.V.D.); paula_ortega31@hotmail.com (P.O.T.); egonrey@ull.edu.es (E.G.R.); 2 Departamento de Ciencias Médicas Básicas, Unidad de Fisiología, Universidad de la Laguna, Tenerife, Canary Islands, 38320 La Laguna, Spain; pabreu@ull.edu.es

**Keywords:** alcoholism, sclerostin, body composition, obesity, Osteosarcopenia, cirrhosis

## Abstract

Background: Sclerostin was initially described as an inhibitor of the Wnt-β catenin bone-forming pathway, but it also exerts important effects on intermediate metabolism and body composition. Osteosarcopenia and altered body fat distribution are common findings in excessive drinkers. The role of sclerostin in these patients is uncertain. We aim to analyze the behavior of sclerostin in excessive drinkers and its relationships with body composition (fat mass, lean mass, bone mass), handgrip strength, body mass index (BMI), liver function and ethanol intake. Methods: 107 male active heavy drinkers and 26 age-matched controls were included. Serum sclerostin was determined by ELISA. Body composition analysis was performed by double X-ray absorptiometry. Handgrip strength was recorded using a dynamometer. Liver function was assessed according to Child’s classification. Results: Sclerostin was higher among Child’s C patients, keeping a relationship with deranged liver function. Obesity, defined according to BMI, and body fat were strongly related to sclerostin, being independent of serum creatinine and of liver function. The relationship of sclerostin with total hip bone mineral density was displaced by BMI. Conclusion: Deranged liver function is associated with higher sclerostin levels in alcoholics. Raised sclerostin levels are related to fat deposition and increased BMI.

## 1. Introduction

Sclerostin is a Wnt-β catenin inhibitor that suppresses bone formation [1]. The binding of Wnt glycoprotein to LRP-5 and to Frizzled (a seven-pass transmembrane co-receptor) triggers a cascade of events that involves several cytosolic proteins and ultimately leads to the accumulation of β-catenin, which acts as a transcription factor for several genes involved in bone synthesis. Binding to LRP-5 is a crucial step in this bone synthetic pathway. Sclerostin binds to LRP-5, thus precluding activation of the Wnt cascade and impairing bone synthesis [2]. Consistent with this effect, sclerostin antibodies have emerged as a useful tool in the treatment of osteoporosis [3]. Some data support the view that sclerostin may also activate bone resorption [4]. Sclerostin may be involved in inflammation-associated bone loss. Indeed, in addition to other effects on osteoclasts and bone homeostasis [5], both tumor necrosis factor (TNF)-α and reactive oxygen species (ROS) upregulate osteocyte sclerostin expression [6]. Despite these actions (inhibition of bone synthesis and promotion of bone resorption), several authors have reported direct relationships between sclerostin and bone mineral density (BMD) or bone mineral content (BMC) [7,8,9,10]. Interpretation of these results is difficult; it has been said that sclerostin levels reflect the osteocyte mass. Possibly, the concomitant presence of confusion factors plays a major role in these paradoxical relationships, since in the last decade it has been shown that sclerostin exerts important effects on intermediate metabolism [11] and on body composition. In this sense, sclerostin levels are related to insulin resistance [12], are higher in diabetics [13] and in patients affected by metabolic syndrome [9], and positively correlate with body fat [14,15], although (as with BMD or BMC) some discrepancy exists regarding this matter. Zhou et al. (2021) found lower sclerostin levels in 168 patients affected by non-alcoholic fatty liver disease [16]—a major feature of metabolic syndrome—in accordance with previous observations reported by Polyzos et al. (2016) in 27 patients with biopsy-proven steatosis or steatohepatitis [17]. Adding controversy, some authors have found an inverse relationship with obesity (i.e., lower values in obese patients [18]), in sharp contrast with the results of Stanik et al. (2019), who showed a direct relationship between sclerostin and obesity in a wide sample of individuals aged less than 18 years [19]; moreover, documented sex differences (higher values in men than in women), both during the growing period [20] and in adult cohorts [21,22] and age-related changes [23] may complicate the interpretation of these findings.

In addition, the Wnt pathway (including also the canonical Wnt- β catenin pathway), is involved in the differentiation of muscle stem cell [24], and muscle fiber hypertrophy is induced by activation of the canonical Wnt-β catenin signaling pathway [25]. Sclerostin is an inhibitor of this pathway and may be involved in sarcopenia [26]. In experimental studies, sclerostin inhibition improves cancer cachexia [27].

Alcoholism constitutes a risk factor for osteoporosis, especially if cirrhosis is also present, both impairing bone synthesis [28] and inducing bone resorption [29]. Ethanol is also a risk factor for altered fat distribution and decreased muscle mass [30,31], and chronic alcoholism is associated with low-grade inflammation [32]. Some reports suggest that sclerostin levels may be influenced by liver function, but results are disparate: some authors report an increase in sclerostin levels among patients with more advanced disease [33,34], whereas others found the opposite [35]. To our knowledge, although relationships among sclerostin and bone variables were thoroughly analyzed in some of these studies, the relationships with fat and lean mass in patients affected by ethanol overuse have been less studied.

Experimentally, it was shown that ethanol enhances sclerostin expression [36], but it was also shown that ethanol promotes osteocyte apoptosis [37]. Therefore, in excessive drinkers, several factors may influence sclerostin levels; this potential alteration may have several effects on bone or intermediary metabolism. It is therefore important to discern the behavior of this molecule in relation to body composition, liver function and variables related to metabolic syndrome in these patients. This is the aim of the present study.

## 2. Materials and Methods

In this cross-sectional observational study, we included 107 male excessive drinkers consecutively admitted to our clinical unit due to organic complications related to alcoholism, although, based on means and standard deviation values of a previous study [33] a minimum of 80 patients (at least 16 controls) would be needed to achieve 85% power with a significance level of 0.05. We included only men given the sex differences reported for sclerostin behavior [22], and the marked differences in body composition between men and women. The mean age was 59.01 ± 10.84 years, like that of 26 healthy male, non-alcoholic hospital workers (54.85 ± 7.71 years, t = 1.83; *p* = 0.06), who served as controls. Assessment of handgrip strength with a Collins dynamometer was performed on 87 patients.

All the patients underwent a complete medical evaluation, including recording of daily ethanol consumption (as volume and type of beverage consumed × grades of alcoholic beverage (%) × 0.8) and years of addiction. Only drinkers of a daily amount >100 g ethanol during at least 5 years were included.

Liver ultrasound (US) examination was performed on all the patients, classifying them as cirrhotics or non-cirrhotics when there were ultrasonographic data of liver affectation (irregular borders, diffuse alteration of the US pattern, and enlarged spleen and/or engorged collateral portosystemic shunts). To achieve a global assessment of liver function we calculated the Child-Pugh’s score considering prothrombin, albumin, bilirubin, and the presence and intensity of ascites and encephalopathy [38,39].

Biochemical parameters: All patients underwent complete routine laboratory analysis. Blood samples were taken at 8.00 am under fasting conditions, in order to determine serum levels of variables related to ethanol consumption such as gamma glutamyl transferase (GGT) and mean corpuscular volume (MCV); liver function variables such as bilirubin, albumin and prothrombin activity; serum creatinine; and variables related to metabolic syndrome, such as total, LDL and HDL cholesterol, triglycerides, uric acid, and glycated hemoglobin. None of them had received albumin or platelet transfusion before blood extraction. Serum sclerostin was determined by ELISA method, using a commercial kit purchased from Thermo Scientific Laboratories (Thermo Fisher Scientific Co., Waltham, MA, USA). The calibration curve of ELISA was set 0–10,000 pg/mL. The assay was evaluated with a 4PL algorithm. The correlation analysis between absorbance units (AU) and standards was 0.9945. The λ max of analysis was established at 450 nm, using a microplate spectrophotometer reader (Spectra MAX-190, Molecular Devices, Sunnyvale, CA, USA). The lower limit of detection (zero + 2 SD) of this assay was 12 pg/mL. Intra and inter-assay coefficients of variation (CV) were 4.32% and 5.18%, respectively. The final serum concentration of sclerostin was expressed in pmol/L (conversion factor: 1 pg/ML = 0.044 pmol/L, molecular weight = 22.5 kDa).

Nutritional assessment: Patients also underwent body composition analysis by densitometry (DEXA), using a HOLOGIC QDR-2000 device (Waltham, MA, USA). We assessed total body fat, lean mass, bone mineral content (BMC) and bone mineral density (BMD), as well as lumbar spine and total hip BMD and total hip and lumbar spine T-score. We also recorded the proportion of fat and lean mass of the android (central) and the gynoid (hip and thigh) regions. We calculated, following Heymsfield et al., the ASM as appendicular lean mass/height, and ASM including only upper limbs and ASM including only lower limbs [40].

Statistical analysis: the Kolmogorov–Smirnov test was used to explore whether the variables were normally distributed, something not fulfilled by sclerostin. Therefore, non-parametric tests, such as Mann–Whitney U test, Kruskal–Wallis and Spearman’s correlation were used. Multivariate analyses were also performed when appropriate, as indicated in the “Results” section. These analyses were performed with SPSS-IBM software (25.0) (Chicago, IL, USA).

The study protocol was approved by the local ethical committee of our hospital (number 2017/50) and conforms to the ethical guidelines of the 1975 Declaration of Helsinki. All the patients gave their written informed consent.

## 3. Results

Sclerostin levels were slightly, non-significantly higher among patients than among controls (Z = 0.49), showing a non-significant trend to higher values among cirrhotics (KW = 3.34; *p* = 0.19). Sclerostin levels were not related to age, but to serum creatinine (ρ = 0.23; *p* = 0.016). Some biological features of cirrhotics and non-cirrhotics are shown in Table 1.

### 3.1. Ethanol Consumption and Markers of Excessive Drinking

No relationships were observed between sclerostin and daily ethanol consumption or years of ethanol consumption (ρ = 0.14; *p* = 0.14 and ρ = 0.18; *p* = 0.07, respectively). We also failed to find any relationship between MCV and serum sclerostin (ρ = 0.07) or between serum sclerostin and GGT (ρ = 0.04; *p* > 0.50 in both cases).

### 3.2. Liver Function

**Figure 1:**
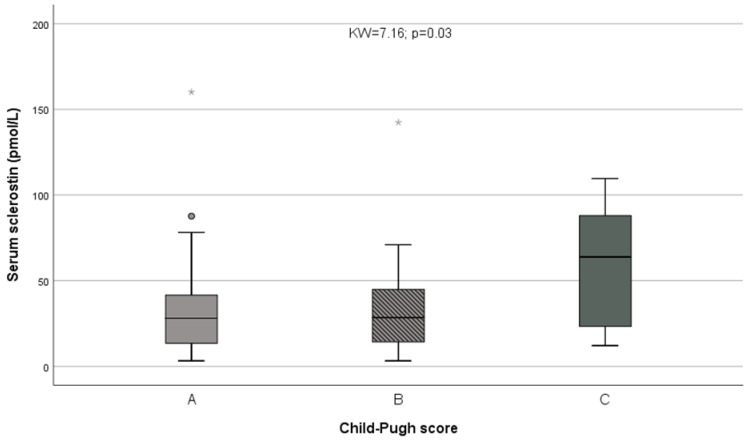
Differences in serum sclerostin levels among Child A, Child B and Child C patients. * = represent extreme values.

Sclerostin showed a non-significant trend to higher values among cirrhotics compared with non-cirrhotics (Z = 1.72; *p* = 0.086). A significant inverse relationship was observed between sclerostin and prothrombin activity (ρ = −0.20; *p* = 0.04); a non-significant inverse correlation between sclerostin and serum albumin (ρ = −0.17; *p* = 0.089), and a non-significant direct correlation with serum bilirubin (ρ = 0.19; *p* = 0.051) were also observed. When liver function was globally assessed using Child’s classification, marked differences were observed in serum sclerostin levels among Child A, Child B and Child C patients (KW = 7.16; *p* = 0.03; Figure 1). Especially, Child C patients showed significantly higher values than those of Child A and Child B patients grouped together (Z = 2.61; *p* = 0.009). In contrast, no relationships were observed between liver function and creatinine (KW = 4.00, *p* = 0.14) among the three Child’s groups.

### 3.3. Body Composition/Nutritional Status

Significant correlations were observed between serum sclerostin and diverse variables related to fat mass, including right leg (ρ = 0.21) and left leg fat mass (ρ = 0.22; *p* < 0.03 in both cases), gynoid fat mass (ρ = 0.29; *p* = 0.004) and android fat mass (ρ = 0.33; *p* = 0.001), trunk fat mass (ρ = 0.19; *p* = 0.045), and total fat mass (ρ = 0.19; *p* = 0.049). Stepwise multiple regression analysis, including the fat variables and serum creatinine, revealed that the first variable selected was android fat mass (beta = 0.74; *p* = 0.001), followed by trunk fat (beta = −0.52; *p* = 0.016), and creatinine (beta = 0.21; *p* = 0.030).

**Figure 2:**
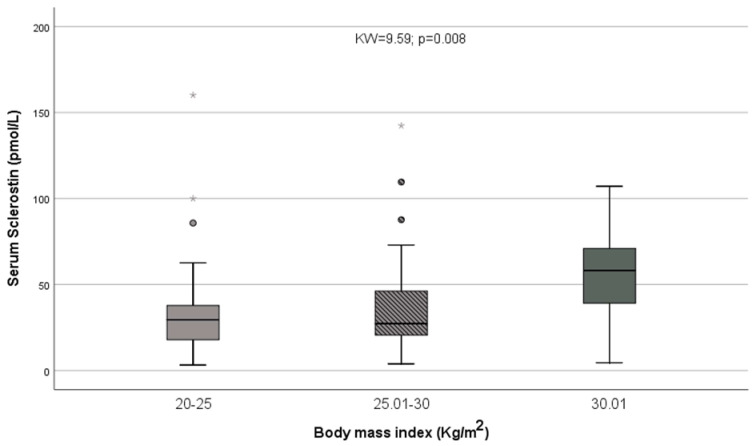
Obese patients (BMI > 30 kg/height^2^) showed higher sclerostin levels than overweighted (BMI between 25.01 and 30 kg/height^2^) and normal patients (BMI between 20 and 25 kg/height^2^). * = represent extreme values.

We also observed a significant relationship between sclerostin and body mass index (ρ = 0.29; *p* = 0.005); this relationship persisted when only patients without ascites were included (ρ = 0.30; *p* = 0.013). Classifying patients according to BMI, obese patients (BMI > 30 kg/height2) showed higher sclerostin levels than overweighted (BMI between 25 and 30 kg/height2) and normal patients (BMI between 20 and 25 kg/height2; KW = 9.59 *p* = 0.008; Figure 2). A similar result was observed when patients were classified as obese or non-obese (Z = 2.94; *p* = 0.003). Results were also significant when patients with ascites were excluded (KW = 8.09; *p* = 0.017 and Z = 2.66; *p* = 0.008, respectively). Creatinine showed a trend to higher values among obese patients vs. overweight and normal individuals (KW = 6.72; *p* = 0.035), but not when patients with ascites were excluded (KW = 5.83; *p* = 0.054); moreover, logistic regression analysis showed that the only variable related to obesity was sclerostin (*p* = 0.023).

There was a significant relationship between sclerostin and trunk lean mass (ρ = 0.20 *p* = 0.044), a relationship that became non-significant if patients with ascites were excluded (ρ = 0.17; *p* = 0.12). On the contrary, the relationship of sclerostin with android lean mass was statistically significant (ρ = 0.21; *p* = 0.037), even if patients with ascites were excluded (ρ = 0.23; *p* = 0.043). Stepwise multiple regression analysis also including creatinine, which showed that sclerostin was the only variable independently related to android lean mass (beta = 0.212; *p* = 0.035). No relationships were observed between sclerostin and handgrip strength (ρ = −0.14; *p* > 0.20), lean mass at upper or lower limbs, ASM, or lower limbs ASM (Table 2). A trend was observed between sclerostin and upper limbs ASM (ρ = −0.20; *p* = 0.055); however, considering only cirrhotics, a trend was observed between sclerostin and handgrip strength (ρ = −0.28; *p* = 0.07).

Sclerostin did not show any relationship with BMC or BMD, besides a direct relationship with total hip BMD (ρ = 0.26; *p* = 0.008); however, a multiple correlation study shows that this relationship became displaced by BMI (beta = 0.26; *p* = 0.014).

### 3.4. Relationships with Hypertension, Diabetes, Serum Lipids and Uric Acid

Patients with hypertension showed a trend to higher sclerostin levels than patients without hypertension. (Z = 1.84; *p* = 0.066). Serum creatinine was also higher among patients with hypertension (Z = 2.67; *p* = 0.009). Multivariate analysis exploring the relationship between hypertension, sclerostin and creatinine by logistic regression showed that the only variable selected was creatinine (*p* = 0.012).

Diabetics also showed a non-significant trend to higher sclerostin values than non-diabetics (Z = 1.51; *p* = 0.13), but no relationship was observed between sclerostin and glycated hemoglobin at admission (ρ = 0.10) or between sclerostin and glycaemia (ρ = 0.09; *p* > 0.35 in both cases). A significant difference in serum creatinine was observed comparing diabetics and non-diabetics (Z = 2.53; *p* = 0.011).

No relationships were observed between sclerostin and any of the variables included in the serum lipid profile (total-, LDL- and HDL-cholesterol, Castelli index, triglycerides), but a significant relationship was observed between sclerostin and uric acid (ρ = 0.30; *p* = 0.01). A strong correlation was observed between creatinine and uric acid (ρ = 0.56; *p* < 0.001), so that the relationship between uric acid and sclerostin disappeared when multiple linear regression analysis was performed, being displaced by creatinine.

Therefore, sclerostin was significantly related to obesity and liver function. There was an association between hypertension and obesity (χ^2^ = 4.73; *p* = 0.03), and also a relationship between hypertension and diabetes (χ^2^ = 5.90; *p* = 0.015), but no relationship between the BMI groups (normal, overweight, obese) and severity of liver disease (Childs A, B or C groups; χ^2^ = 1.01; *p* = 0.60); however, in order to disclose which of these variables were independently related with sclerostin levels, we classified sclerostin according to median values and performed a logistic regression analysis introducing the variables cirrhosis, obesity, hypertension and diabetes. The only variable which showed an independent relationship with sclerostin was obesity (*p* = 0.015). The same happened when the variable Child’s group was introduced. When the variable creatinine (over/below the median) was introduced, both creatinine (*p* = 0.001) and obesity (*p* = 0.024) were independently related to sclerostin over or below the median, but when a multiple regression analysis between sclerostin (as the independent variable) and creatinine and BMI was performed, only BMI -but not creatinine- was independently related to sclerostin (beta = 0.26; *p* = 0.014).

## 4. Discussion

This study was performed to explore the behavior of serum sclerostin in excessive drinkers, especially in relation to liver function derangement and altered body composition. The main results of this study show that serum sclerostin increases in relation to liver function impairment and that there is an independent direct relationship of sclerostin levels with obesity and fat mass, especially gynoid and android fat mass. These relationships are independent on serum creatinine, a variable strongly related to serum sclerostin.

The relation of sclerostin with fat and obesity is in accordance with the current knowledge about the effects of sclerostin on intermediate metabolism. In addition to its inhibitory effect on the Wnt-β catenin-dependent bone-forming pathway, sclerostin exerts many other actions, constituting a major example of the so-called osteokines, a group of molecules that govern the interplay of the bone-intermediate metabolism.

Although with some exceptions [16], both clinical [9,14,15,41] and experimental studies support a direct effect of sclerostin on adipogenesis. In vitro studies have shown that sclerostin promotes differentiation of preadipocytes into mature adipose cells [42], increases fatty acid synthesis and reduces fatty acid catabolism in these cells [11]. Circulating sclerostin levels are associated with higher vertebral marrow fat in men, suggesting a relationship between osteocyte function and marrow adipogenesis [43]. Sclerostin gene knockout animals showed a markedly reduced fat mass and reduced adipocyte size and bone marrow adipose tissue, whereas in a high circulating sclerostin model, an increased white adipose tissue was observed [44].

In the present study, sclerostin levels showed a trend (but not significant) to higher values among cirrhotics, and a significant relationship with liver function derangement, with higher levels being observed among Child C patients. The behavior of sclerostin in liver cirrhosis has been studied by some researchers, yielding disparate results. Wakolbinger et al. (2020), in a series of 32 cirrhotic patients, found non significantly, slightly lower sclerostin values—especially among the 16 alcoholics included in the study, among whom the decrease in sclerostin levels was statistically significant; these authors also describe a significant relationship with liver function (MELD score) and, as in the present study, with total hip BMD [35]. In another study dealing with cirrhotic patients (mainly viral cirrhotics), Rhee et al. (2013) found higher sclerostin levels in patients with more deranged liver function [34]. In a preliminary report on 31 excessive drinkers, sclerostin was also higher among patients with more severe liver disease [33], and in a series of 40 hepatitis C virus-infected patients with higher BMD values than controls we showed that sclerostin values were similar in patients than in controls and were directly related with BMD. Although in that study body fat was not assessed, sclerostin did show a significant relationship with histomorphometrically assessed liver fat, although not with Knodell index or liver function, besides a direct, borderline significant relationship with serum bilirubin (*p* = 0.046) [45]. On the contrary, Zhou et al. (2021), in 168 patients, found lower sclerostin levels than in controls, and a negative correlation with the fatty liver index (calculated from serum triglycerides, GGT, BMI and waist circumference [16]). Therefore, there is controversy regarding the relationship with liver function, but in our study, it seems that sclerostin increases with deranged liver function, in accordance with Rhee et al. (2013) [34], and with previous results [33]. Our data are in accordance with the hypothesis of Rhee et al. (2013) that liver plays some role on sclerostin clearance [34], a hypothesis that received recent scientific support by the finding of Jørgensen et al. (2021) [46], who reported that hepatic sclerostin extraction was lower in 59 cirrhotics (47 alcoholics), strongly suggesting that sclerostin is eliminated through the liver and explaining the raised levels described in cirrhotics. Given the apparently suppressive effect of estrogens on serum sclerostin levels [22,47] the altered hormonal profile (increased estrogen/androgen ratio) described in cirrhotics might also play a role.

As stated, in agreement with the invitro and experimentally documented information, we did find a significant relationship between fat amount or obesity and sclerostin levels. The relationship with obesity explains the paradoxical result of a direct correlation with total hip BMD, because, at least in our study, this relationship depends, in fact, from increased BMI. Paradoxical direct relationships between sclerostin and BMD or BMC have been reported by several authors [7,48], and several explanations have been argued, including an increased osteocyte mass among those with increased BMD [8] (although in that study it was also reported that a low BMD among those with the highest sclerostin quartile implies an increased risk of fracture [8]), or a confounding effect of fat mass [10], or problems related with sclerostin assay [49].

Other authors explored the relationship of sclerostin with muscle mass. Kim et al. (2019) examined 240 healthy non-diabetic subjects, and found an inverse, significant association between sclerostin and ASM (sclerostin higher in individuals with low muscle mass [50]). On the contrary, Moriwaki et al. (2019) found a direct relationship between grip strength and sclerostin (r = 0.309; *p* < 0.01), between BMI and sclerostin, and between ASM and sclerostin in a population-based study including middle-aged and elderly individuals [51]. In a previous study, we found an inverse correlation between handgrip and serum sclerostin among 59 patients affected by ethanol overuse [52]. The results here reported agree with the previous observations, and the commented effects of sclerostin on muscle [26], although, strikingly, they are restricted to cirrhotics, perhaps in relation with the higher values of sclerostin observed in this group both compared with non-cirrhotic patients and controls. Medeiros et al. (2020), in 41 diabetic patients and 51 non-diabetics, with end-stage kidney failure, also report an inverse relationship between sclerostin and handgrip [53]. Thus, although some discrepancy exists, it seems that, overall, raised sclerostin levels may be involved in sarcopenia, something that was also observed in the alcoholic cirrhotics included in this study. Therefore, the raised sclerostin levels found in cirrhotics in the present study may be involved in some of the systemic alterations described in these patients. Besides the association with liver function derangement (that should be confirmed, given the disparate results commented before) and the possible relation with myopathy, the relationship with fat mass may be of particular concern. Obesity is a well-known cardiovascular risk factor, but a relationship between increased sclerostin levels and vascular risk has also been recently reported [54]. Although controversy exists regarding the vascular effects of sclerostin [55], and although in this study the relationship between sclerostin and hypertension was displaced by creatinine, undoubtedly, more research exploring the behavior of sclerostin in cirrhotics, confirming its relation with obesity, and exploring its relationship with mortality and/or vascular events are necessary. A possible benefit of a therapeutic approach with antisclerostin antibodies in these patients should be also tested; moreover, similar studies should be performed on women affected by alcoholism.

## 5. Conclusions

We conclude that deranged liver function leads to raised sclerostin levels in men affected by alcohol overuse; these raised sclerostin levels are related to obesity and gynoid and android fat accumulation. It can be speculated that altered hepatic clearance of sclerostin by the cirrhotic liver leads to raised sclerostin levels, that, in turn, may be involved in the altered body composition described in these patients.

## Figures and Tables

**Table 1:** Some biological features of cirrhotics and non-cirrhotics.

	Cirrhotics (*n* = 54)	Non-Cirrhotics (*n* = 53)	T (Z); *p* *	Controls (*n* = 26)
Age	59.91 ± 10.40	58.09 ± 111.49	T = 0.86; NS	54.85 ± 7.71
Daily ethanol (g)	203 ± 110 200 (139–250)	218 ± 180 200 (100–269)	Z = 0.18; NS	<10 g
Years of addiction	32.56 ± 13.15	32.82 ± 13.13	T = 0.11; NS	-
Mean corpuscular volume (fL)	101.29 ± 10.55	101.04 ± 5.29	T = 0.16; NS	92.49 ± 4.31
Serum GGT (U/L)	374.67 ± 658.68 208.00 (54.00–396.50)	229.90 ± 252.96 116.00 (59.50–345.00)	Z = 0.84; NS	26.39 ± 10.77
Prothrombin activity (%)	66.31 ± 21.28 63.50 (50.75–83.75)	89.79 ± 12.37 95.00 (84.00–100.00)	Z = 5.59; *p* < 0.001	97.50 ± 4.15
Serum Albumin (g/dL)	3.45 ± 0.78	3.75 ± 0.62	T = 2.19: *p* = 0.031	4.56 ± 0.24
Serum bilirubin (mg/dL)	3.61 ± 4.28 2.30 (1.00–4.35)	1.40 ± 1.88 1.00 (1.00–1.08)	Z = 5.45; *p* < 0.001	0.99 ± 0.11
Serum sclerostin (pmol/L)	41.19 ± 30.52 33.73 (16.32–59.95)	31.48 ± 26.93 28.05 (12.90–40.30)	Z = 1.72; *p* = 0.086	29.14 ± 13.52
Serum creatinine (mg/dL)	1.02 ± 0.73 0.72 (0.62–0.95)	0.81 ± 0.28 0.76 (0.61–0.95)	Z = 0.06; NS	0.91 ± 0.11
Serum cholesterol (mg/dL)	124.36 ± 36.22	172.28 ± 49.53	T = 5.58; *p* < 0.001	188.94 ± 40.01
Serum triglycerides (mg/dL)	92.06 ± 38.39	123.72 ± 59.98	T = 3.17; *p* = 0.002	108.39 ± 46.19
Serum uric acid (mg/dL)	5.84 ± 2.54	5.17 ± 2.55	T = 1.09; NS	5.46 ± 0.76
Glycated hemoglobin (%)	5.93 ± 1.74 5.40 (4.98–6.15)	5.46 ± 0.51 5.40 (5.08–5.63)	Z = 0.39; NS	5.79 ± 0.64
Total HIP BMD	1.028 ± 0.163	1.012 ± 0.197	T = 0.88; NS	1.05 ± 0.14
Pelvis BMD	1.143 ± 0.122	1.105 ± 0.151	T = 1.41; NS	-
Total BMD	1.186 ± 0.106	1.186± 0.122	T = 0.02; NS	-
L2-L4 BMD	1.162 ± 0.165	1.143 ± 0.208	T = 0.50; NS	1.04 ± 0.06
Total fat (g)	22,733 ± 11,167	19,733 ± 9505	T = 1.75; *p* = 0.084	21,329 ± 7059
Trunk fat (g)	13,398 ± 6155	11,682 ± 6252	T = 1.71; *p* = 0.091	11,280 ± 4851
Right leg fat (g)	3334 ± 2022	2752 ± 1415	T = 1.93; *p* = 0.057	3589 ± 1032
Left leg fat (g)	3295 ± 1970	2741 ± 1393	T = 1.87; *p* = 0.064	3550 ± 1005
Right arm fat (g)	1054 ± 723	884 ± 473	T = 1.61; NS	1214 ± 510
Left arm fat (g)	1008 ± 652	938 ±486	T = 0.82; NS	1150 ± 472
Gynoid fat (g)	3753 ± 2045	3083 ± 1570	T = 1.83; *p* = 0.070	-
Android fat (g)	2351 ± 1195	2095 ± 1176	T = 1.07; NS	-
Total lean (g)	50,513 ± 8286	48,753 ± 7271	T = 1.48; NS	54,933 ± 7100
Trunk lean (g)	26,279 ± 4839	24,536 ± 3612	T = 2.29; *p* = 0.024	27,467 ± 3335
Right arm lean (g)	2661 ± 547	2763 ± 654	T = 0.43; NS	3323 ± 556
Left arm lean (g)	2657 ± 608	2774 ± 710	T = 0.51; NS	3163 ± 529
Right leg lean (g)	7639 ± 1690	7378 ± 1449	T = 1.17; NS	8661 ± 1097
Left leg lean (g)	7557 ± 1693	7351 ± 1374	T = 1.00; NS	8363 ± 1268
Gynoid lean (g)	6470 ± 1494	6245 ± 1670	T = 0.71; NS	-
Android lean (g)	4343 ± 1086	3723 ± 927	T = 3.06; *p* = 0.003	-
BMI (kg/m^2^)	26.70 ± 4.91	25.95 ± 4.45	T = 0.76; NS	26.22 ± 2.58
Handgrip strength (kg)	15.40 ± 10.88	27.93 ± 46.99	Z = 1.35; NS	40.21 ± 9.18

* Comparing cirrhotics and non-cirrhotics. We show mean values ± SD, and median values and interquartile range for those variables that did not show a parametric distribution. In the last column right, we also show the data of the controls.

**Table 2:** Relationships between serum sclerostin and lean mass in all patients and considering only cirrhotics.

	Serum Sclerostin	
	All Patients (*n* = 107)	Cirrhotics (*n* = 54)
Lean mass at upper of lower limbs ASM *	ρ = −0.03; NS	ρ = −0.01; NS
Upper limbs ASM	ρ = −0.20; *p* = 0.055	ρ = −0.23; NS
Lower limbs ASM	ρ = 0.01; NS	ρ = 0.04; NS
Handgrip strength	ρ = −0.14; NS	ρ = −0.28; *p* = 0.07

* ASM = appendicular lean mass/height.

## Data Availability

The data presented in this study are available on request from the corresponding author.
